# Evidence that hPIV2 paramyxovirus antigenomes are edited during infection

**DOI:** 10.1128/mbio.03667-24

**Published:** 2025-06-24

**Authors:** Keisuke Ohta, Junna Kawasaki, Daniel Kolakofsky, Machiko Nishio, Yusuke Matsumoto

**Affiliations:** 1 Department of Microbiology, School of Medicine, Wakayama Medical University 13145, Wakayama, Japan; 2 Graduate School of Medicine, Chiba University 12737, Chiba, Japan; 3 Department of Microbiology and Molecular Medicine, University of Geneva School of Medicine 218785, Geneva, Switzerland; 4 Transboundary Animal Diseases Research Center, Joint Faculty of Veterinary Medicine, Kagoshima University 12851, Kagoshima, Japan; Boston University Chobanian & Avedisian School of Medicine, Boston, Massachusetts, USA

**Keywords:** paramyxovirus, viral replication, rule of six, RNA editing

## Abstract

Mononegavirus genomes contain *cis*-acting sequences that direct the formation of mRNA 5′ and 3′ ends during synthesis (gene start [GS] and gene end [GE]) but are silent during antigenome synthesis from the same genome template. Paramyxo- and filoviruses also carry an additional *cis*-acting sequence (EDIT) that directs the insertion of specific nucleotide during mRNA synthesis, expanding gene products. This “mRNA editing” is associated with viruses whose replication is governed by the rule of six. One might, therefore, assume that EDIT, like GS and GE, would be restricted to that of mRNA synthesis. However, accurate determination of the ratio of edited to unedited RNAs in different sub-populations of wild-type and mutant human parainfluenza virus type 2 (hPIV2) infections finds evidence that antigenomes are also edited during infection. This alters our view of how the rule of six governs mononegavirus infections and why this rule, RNA editing, and bipartite promoters are linked.

**IMPORTANCE:**

We have assumed that paramyxovirus editing signals would operate strictly during mRNA synthesis, as it apparently makes no sense to edit antigenomes. Nevertheless, there is evidence here that the opposite is the case. If so, this alters our view of paramyxovirus replication, and we summarize what is known about how its RNA-dependent RNA polymerase carries out its task of expressing alternate open reading frames during mRNA synthesis.

## OPINION/HYPOTHESIS

Non-segmented negative-strand RNA virus (mononegavirus) genomes are 12–21 kilobases long and contain 5–10 tandemly arranged genes. There are four main families: *Rhabdoviridae*, *Pneumoviridae*, *Paramyxoviridae* (Sendai virus [SeV]), human parainfluenza virus type 2 [hPIV2]), and *Filoviridae* (Ebola virus [EBOV]). These genome RNAs are assembled within a non-covalent homopolymer of the nucleoprotein (NP) to form helical nucleocapsids (NCs), the substrate for viral RNA synthesis. The NP subunits (protomers) of each family bind a distinct number of nucleotides (nt); paramyxo- and filovirus NPs bind precisely 6 nt, whereas those of pneumo- and rhabdoviruses bind 7 and 9 nt/protomer, respectively (1). The structure of the genome RNA within each NP RNA-binding groove is also specific to each family; for both paramyxo- and filoviruses, 3 contiguous nt point toward the protein core, and 3 point toward the solvent where they can more readily interact with the viral polymerase (pol) (2, 3).

**Fig 1:**
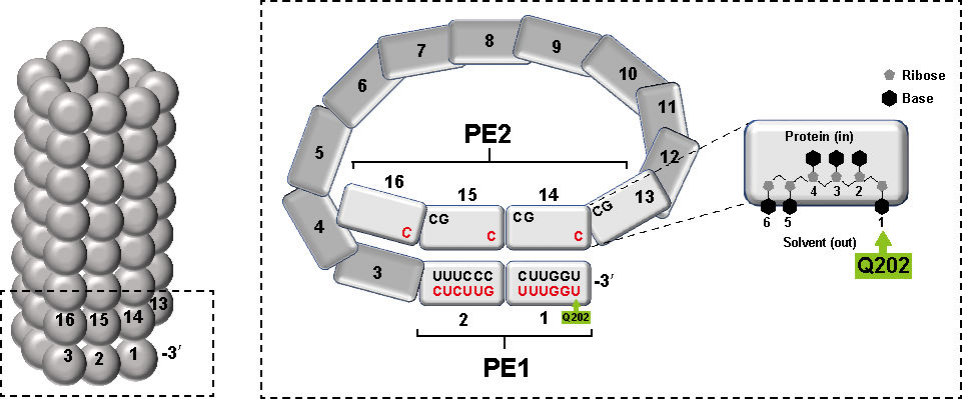
Paramyxovirus bipartite promoter model of the first turn of the NC helix with 13 subunits per turn. The NP subunits are shown as rectangles numbered from the 3′-OH end, each binding precisely 6 nt. The relevant nt sequences of promoter element 1 (PE1) at the 3′ end and the tripartite PE2 in the NP subunits on the next helical turn (and whose bases point toward the solvent) are shown for hPIV2 (black) and SeV (red). Q202 of each subunit can contact the 3′-most base of each hexa-nt, including the genome’s very 3′ uridine, and this last interaction is proposed to prevent NP dissociation from PE1, a prerequisite for its entry into the synthesis chamber in pol’s core for RNA synthesis to initiate. When glutamine is present at position 202, RNA synthesis can initiate, but only if pol can simultaneously interact with a properly aligned tripartite PE2 to reverse the Q202-3′ uridine interaction.

The entire RNA within SeV NCs, including its very ends, is resistant to RNase A cleavage by the assembled NP (4). As each NP binds precisely 6 nt, NCs can be viewed as a succession of protomers each associated with a hexa-nt. If this NC assembly starts at the very 5′ end of the nascent chain and assembly continues 6 nt at a time, the NC would be composed of a succession of protomers, each associated with a hexa-nt whose relative nt orientations within each RNA-binding groove (contiguously stacked triplets pointing in or out) would be the same (Fig. 1), which has been similarly reported for NCs of measles virus (MeV) (5). This “hexamer phase” would be maintained throughout the NC and is known to affect several aspects of SeV mRNA synthesis (6).

Paramyxoviruses have been known to follow the so-called rule of six (7). This term was coined when minigenome studies found that only SeV genomes that are precisely a multiple of 6 nt in length (6*n* + 0) can be replicated (7). Furthermore, their promoters that direct RNA synthesis are bipartite in nature: the first element within the leader region (promoter element 1 [PE1]) and PE2 within the untranslated region (UTR) of the first gene (Fig. 1) (8, 9). Curiously, the sequence of the spacer region in between is not important, even though it includes the transcription start site. However, its precise length is apparently critical, as this region can tolerate nt insertions or deletions only if they involve 6 nt, or multiples thereof. This requirement presumably results from the need to maintain a given hexamer phase between PE1 and PE2 for RNA synthesis to initiate, as pol must interact with both promoter elements for synthesis to initiate. Filoviruses, like paramyxoviruses, also contain bipartite promoters, and maintaining their relative hexamer phases of the promoter elements also appears to be important (9). This analogous spacer region cannot tolerate a single nt difference between PE1 and PE2 (10).

Recent work with hPIV2 has provided insight into the nature of bipartite promoters. Glutamine at NP position 202 is the only residue of its RNA-binding groove that contacts an nt base rather than the ribose-phosphate backbone (11). When mutated to alanine (Q202A) or other residues, minigenome replication is no longer governed by the rule of six—minigenomes of any length can be replicated, and the precise spacer length becomes much less important. NP^Q202A^ is thus a loss-of-function mutation and is positioned to interact with the most 3′ nt of each hexa-nt, including the genome’s very 3′ uridine (U^3′OH^) (Fig. 1) (11). This interaction could prevent the 3′ terminal NP(s) from dissociating from the genome RNA’s 3′ end, a prerequisite for the genome RNA entering the synthesis chamber within pol’s core via the narrow template entry channel. In this view, PE1 is a negative promoter element controlled by this Q-U^3′OH^ interaction, and PE2 functions to relieve this inhibition so RNA synthesis can begin if the relative hexamer phases of PE1 and PE2 have been maintained. In the absence of this negative element (e.g., Q202A), the restraints of the rule of six are apparently relieved.

Paramyxo- and filoviruses also share another property: co-transcriptionally edit an mRNA to expand the number of gene products (9, 12). Paramyxoviruses like SeV or MeV add a single guanidine (G) to switch from the P to V open reading frames (ORFs), whereas others like hPIV2 and PIV5 insert two Gs to switch ORFs from V back to P (13–16). Further viruses, like bovine PIV3 (bPIV3) or Nipah virus (NiV), add a variable number of nt at the editing site (EDIT) during mRNA synthesis to accommodate the expression of another P gene protein, W (17, 18). Rhabdo- and pneumoviruses, in contrast, do not edit their mRNAs, do not contain bipartite promoters, and share no aspects of a “rule of any integer.” Interestingly, EBOV that edits its receptor binding protein (RBP) mRNA (via an adenosine insertion to express full-length RBP) (19, 20) is known to sometimes also insert the same adenosine during antigenome synthesis, i.e., to “edit” its antigenome (21). Whether this occurs during paramyxovirus infection has not been reported and is the subject of this study.

### The rule of six and genome replication

**Fig 2:**
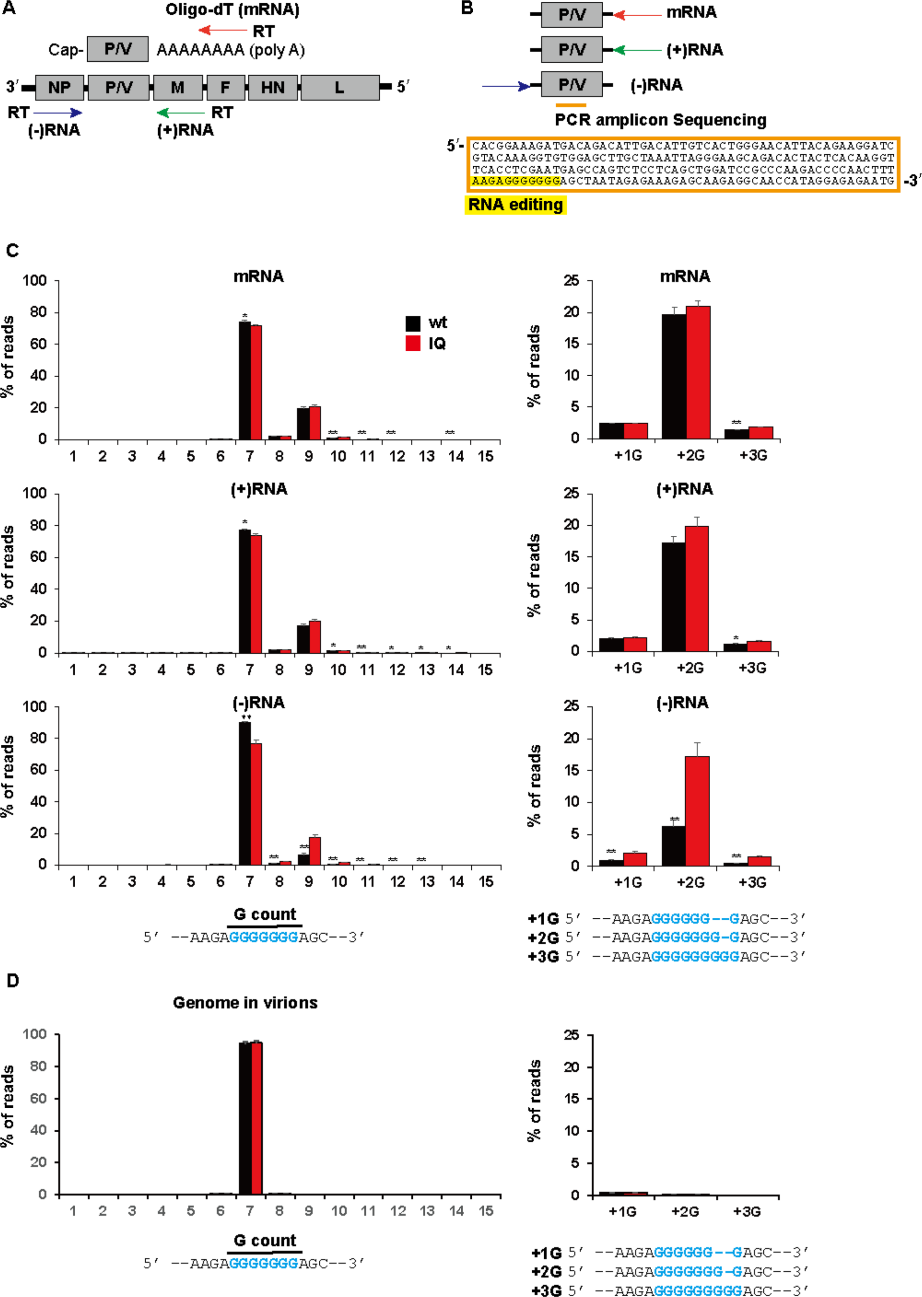
Amplicon sequencing of the editing region of hPIV2 P/V mRNA, viral (+)RNA and (−)RNA. (**A**) Schematic diagram of the hPIV2 genome and primers for reverse transcription of each viral RNA in wild type (wt) and IQ-infected Vero cells. (**B**) Scheme for the reverse transcription reaction. PCR amplification was performed for the area containing the P/V gene editing region in mRNA, (+)RNA, and (−)RNA. The sequence of the PCR amplicon is shown, and the RNA editing region is highlighted in yellow. (**C**) G count in viral RNAs within the infected cells; the number of guanosines in the RNA editing region (left panel) and the magnified graph (right panel). G + 1, 2, and 3 indicate the number of additional Gs over the unedited seven Gs. (**D**) G count of the viral genome within the purified progeny virions. Data represent means and standard deviations (*n* = 3). *P < 0.05 and **P < 0.01 (wt vs IQ, a two-tailed unpaired Student’s *t*-test).

To examine the editing status of hPIV2 RNAs, Vero cells were infected with rPIV2^wt^ or rPIV2^IQ^ at a multiplicity of infection (MOI) of 0.1, and total RNA was extracted at 48 hpi. The nucleoprotein of rPIV2^IQ^ carries two mutations: Q202A, which relieves the rule of six, and I35L, which is required for genomes that carry Q202A to form virus progeny (11, 22). cDNA for mRNA was generated by reverse transcription using oligo-dT (Fig. 2A and B). cDNA for genome RNAs [(−)RNA] was generated with a primer from the upstream NP gene, and cDNA for plus-strand viral RNAs [(+)RNA], including antigenomes and P-M readthrough mRNAs, was generated with a primer from the downstream M gene (Fig. 2A and B). PCR was performed using these cDNAs as templates to amplify a 212 nt region spanning EDIT (Fig. 2B). The PCR products were subjected to amplicon sequencing, and approximately 200,000 copies of each amplicon were examined. We can thus accurately determine the percentage of edited vs non-edited RNAs in each RNA population (Fig. 2C).

As our results are read from dsDNA, only the “G count” is recorded, independent of the origin of the insertion. The hPIV2 genome editing site (3′ …UUCUCCCCCCC… 5′) contains a run of seven cytidines that are copied into a run of nine guanidines at a given frequency during mRNA synthesis (19). In both rPIV2 infections, (+2G)mRNAs represented about 20% of the total mRNAs, and the vast majority of the remainder were unedited (Fig. 2C, mRNA), consistent with our previous study (11). When (+)RNAs in the subpopulation generated with the downstream M gene primer were examined, very similar results were unexpectedly found. As this methodology does not distinguish between antigenomes and readthrough mRNAs retaining the P-M junction, we do not know the relative proportions of each of these (+)RNAs in this sub-population. Moreover, we cannot directly compare their relative proportions by quantitative real-time RT-PCR (RT-qPCR) without knowing the relative primer efficiencies.

**Fig 3:**
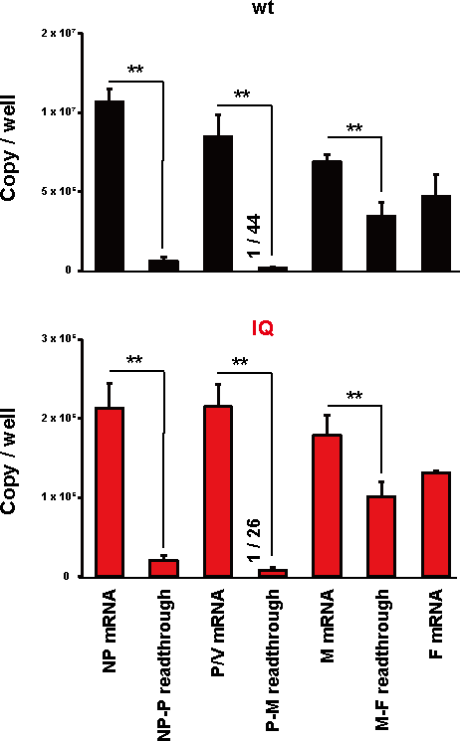
Quantification of NP, P/V, M, F mRNAs and NP-P, P-M, M-F readthrough mRNAs in virus-infected cells. Vero cells were infected with rPIV2^wt^ or rPIV2^IQ^ at an MOI of 0.1 for 48 h. Total RNA was extracted from the infected cells, and copy numbers of the NP, P/V, M, F mRNAs and NP-P, P-M, M-F readthrough mRNAs were measured by RT-qPCR. Data represent means and standard deviations (*n* = 3). **P < 0.01. A comparison between P mRNA and P-M readthrough mRNA in rPIV2^wt^ and rPIV2^IQ^ reveals ratios of 1/44 and 1/26, respectively.

However, we can directly compare the levels of mono- and di-cistronic mRNAs generated with the same oligo-dT primer and use that as a guide. The RT-qPCR results of the first three genes (Fig. 3) are consistent with previous reports for hPIV2/PIV5 infection (23). Other than the M gene, which is known to have a high proportion of M-F readthrough mRNAs, that of the P gene is the lowest. By RT-qPCR, there are 44 times more P mRNA than P-M readthrough mRNA in wild-type (wt) infection and 26 times more in IQ infection. The unexpected result mentioned above is that, despite the RT-qPCR results, the fraction of +2 nt-inserted RNA in this sub-population virtually mirrors that of polyadenylated mRNAs (Fig. 2C). Since the relative amounts of antigenomes and P-M mRNAs are unknown in our infections, we cannot exclude the possibility that all the +2 nt-inserted RNAs here are due to readthrough mRNAs, even if this seems unlikely given that P-M mRNAs represent such a small fraction of the polyadenylated RNAs. For example, if there were equal amounts of antigenomes and P-M mRNAs in this sample and the latter contained the same level of 2 nt-inserted RNA as P mRNAs, the fraction of +2 nt-inserted RNA would be half that of P mRNAs, a difference that is well within the margin of error of this method. Rather, given the method’s sensitivity, it appears that antigenomes may be edited as frequently as mRNAs.

There is also genetic evidence that antigenomes are edited during infection. Six percent of the RNA in the genome subpopulation in the wt infection has the same 2 nt insertion. Moreover, whereas the presence of NP^IQ^ during infection has little or no effect on the levels of edited polyadenylated RNAs or edited (+)RNAs (Fig. 2C), NP^IQ^ has a strong effect on the level of 2 nt-inserted RNA [presumably, pre-edited (+2C) genomes], which are now three times as abundant in the IQ infection as in the wt infection (Fig. 2C). These results are consistent with some (+2G) antigenomes being generated in wt infection, but which are relatively poor templates for replication to (+2C) genomes, as the rule of six acts in part at the initiation of genome replication. When this rule is relieved in the IQ infection, (+2G) antigenomes (6*n* + 2 nt) become better templates for replication to (+2C) genomes. Thus, the available evidence, although indirect, indicates that antigenomes are indeed edited during hPIV2 infection.

### The rule of six and virus maturation

This study was motivated by questioning whether hPIV2 genomes that are not of hexamer length could replicate at all during a wt infection. The above results suggest that (+2G) antigenomes are generated during infection and copied into (+2C) genomes, albeit with less efficiency than their 6*n* + 0 counterparts. To determine whether this reduced efficiency is sufficient to maintain the rule of six, we examined the editing status of hPIV2 RNAs in culture supernatants at 48 hpi (Fig. 2D). Supernatant virions were concentrated by pelleting through a glycerol cushion and examined as above. We confirmed minimal amplification of antigenomes or mRNAs in the virion preparation; thus, only the genome amplicon was subjected to sequencing. Remarkably, independent of the fraction of (+2C) genomes intracellularly at 48 hpi (6% in the wt infection or 17% in the IQ infection), only 6*n* + 0 genomes were present in the supernatant (Fig. 2D).

### Intracellular hPIV2 genome replication

Our results can be interpreted in two ways. Either readthrough mRNAs are so abundant relative to antigenomes that all (+2G)(+) strands are due to P-M readthrough mRNAs, with no editing of antigenomes. Or both antigenomes and P-M readthrough mRNAs contain the same (+2G) insertion, i.e., at least some antigenomes are edited, possibly as frequently as mRNAs. Our results support only the latter, and in this case, our view of how the rule of six impacts paramyxovirus replication needs to be reexamined.

All mononegavirus genomes contain *cis*-acting sequences that signal gene start (GS) and gene end (GE). Clearly, GS and GE’s functioning must be restricted to that of mRNA synthesis. These signals are somehow ignored during antigenome synthesis, and it was reasonable to assume that EDIT would be similarly regulated. GE is of particular interest here as mRNA poly A tails are thought to be formed by pol “stuttering” on a short run of template uridines within GE by pol backtracking on the template, resulting in a pseudo-templated nt insertion upon continued elongation; a cycle when repeated eventually leads to mRNA release from the transcription complex. Despite the differences between GE and EDIT, the latter nevertheless presumably also carries out this process by pol backtracking on a “slippery” template sequence and pseudo-templated nt insertion (24). This process presumably does not occur at the various GEs during antigenome synthesis when product RNA’s elongation appears to be coupled to its assembly with NP, but the same should be true for EDIT. Paramyxovirus EDIT is a true *cis*-acting signal; when added to the SeV L gene 3′ UTR, it ectopically directs mRNA editing very similarly to that within the P gene, and even its hexamer phase impacts the process (6). Thus, one might have expected that whatever mechanism prevents pol stuttering at GEs during antigenome synthesis would also operate at EDIT. This study, however, finds that this is not the case; EDIT appears immune to the anti-stuttering mechanism that operates at GE during genome replication.

Although GE and EDIT may share a common mechanism for nt insertions, they differ significantly. Paramyxovirus EDIT is extremely variable and adaptable to each virus’s needs. hPIV2 RNA editing stands at one end of this variability spectrum. hPIV2 RNA editing specifically adds two Gs when insertions occur, and insertions occur only once or not at all (Fig. 2C). Similarly, SeV requires a single nt insertion to switch ORFs, and SeV inserts only a single nt when insertions occur, and insertions again appear to occur only once or not at all (25). In contrast, the closely related bPIV3 adds 1 to 6 nt at roughly equal frequency when insertions occur to accommodate the expression of an additional P gene product (W) (18). At the other end of this variability spectrum is NiV, which also expresses a W protein: 82% of its transcripts are edited, with up to 11 G insertions observed (17). mRNA polyA tail formation, in contrast, requires none of this flexibility, only that it be restricted to mRNA synthesis.

**Fig 4:**
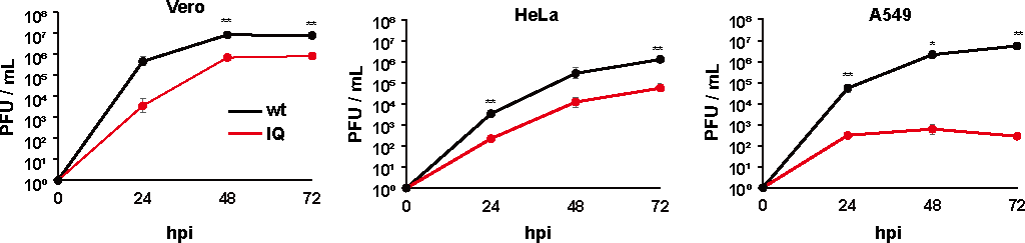
Growth of rPIV2^wt^ and rPIV2^IQ^ in Vero, HeLa, and A549 cells. Vero, HeLa, and A549 cells were infected with rPIV2^wt^ or rPIV2^IQ^ at an MOI of 0.1. The supernatants were then sampled at various times post-infection and titered on Vero cells. Data represent means and standard deviations (*n* = 3). *P < 0.05 and **P < 0.01 (wt vs IQ, a two-tailed unpaired Student’s *t*-test).

Paramyxoviruses express their essential pol cofactor P and non-essential anti-host defense factor V from the same cistron via RNA editing, regardless of which is directly genome-encoded. However, if (+2G) antigenomes are accumulated during hPIV2 infection and generate (+2C) genomes, the latter’s mRNAs would express P rather than V. Diminishing V expression relative to P would render the infection more sensitive to the host antiviral response. In this view, one way in which RNA editing and the rule of six are linked is because the latter is needed to control the negative effects of the former, i.e., to limit altering the ratio of gene products during infection. The relative inability of rPIV2^IQ^ to grow in the more immunocompetent A549 cells (Fig. 4) may be evidence of the negative effects of altering the ratio of P gene products.

Another way RNA editing and the rule of six are linked is at the level of virus maturation. Independent of the proportion of non-6*n* + 0 genomes present intracellularly or whether the rule of six is operative during genome replication, non-6*n* + 0 genomes are effectively excluded during the budding process (Fig. 2D). The rule of six for hPIV2 would then be maintained both by the relative inefficiency of non-6*n* + 0 genome replication and the discrimination against the inclusion of non-6*n* + 0 genomes during maturation. If the latter is a general property of paramyxoviruses, this could explain how viruses like NiV can tolerate such a remarkable assortment of edited RNAs during infection and still remain precisely of hexamer length.

## Data Availability

All study data are included in the article and/or supplemental material. The data set of amplicon sequencing is available in the DNA Data Bank of Japan (DDBJ) under run accession numbers DRR524078–DRR524095 and DRR668219–DRR668224.

## SUPPLEMENTAL MATERIAL

Supplemental material mbio.03667-24-s0001.docx Materials and methods used in this study.

ASM does not own the copyrights to Supplemental Material that may be linked to, or accessed through, an article. The authors have granted ASM a non-exclusive, world-wide license to publish the Supplemental Material files. Please contact the corresponding author directly for reuse.
