# Supplementary material for: Evidence that hPIV2 paramyxovirus antigenomes are edited during infection
Source: mBio. 2025 Jun 24;16(8):e03667-24. doi: 10.1128/mbio.03667-24 (PMC12345181; doi:10.1128/mbio.03667-24)
Supplement: Supplemental material — Materials and methods used in this study. [file mbio.03667-24-s0001.docx]

**Materials and Methods**

**Cells and viruses**

Vero cells were cultured in Eagle's minimal essential medium supplemented with 10% FCS at 37°C in 5% CO_2_. HeLa and A549 cells were cultured in Dulbecco's modified Eagle's medium supplemented with 10% fetal calf serum (FCS) at 37°C in 5% CO_2_.rPIV2^wt^ and rPIV2^IQ^ were generated as previously reported (1).

**RT-PCR for the RNA editing region of viral RNAs**

The Vero cells were infected with rPIV2^wt^ or rPIV2^IQ^ at an MOI of 0.1 and total RNA in the infected cells was extracted at 48 hpi using Isogen II (Nippon Gene, Tokyo, Japan) according to the manufacturer’s instructions. cDNA synthesis was carried out using a PrimeScript RT reagent kit (Takara, Shiga, Japan) with oligo-dT as a specific primer for mRNA; 5'-TCTTGAGCTAGTTGGAGATTTTGATTC-3' for viral (-)RNAs, and 5'-CTGGTAATGATATTATAGGCATGTC-3' for viral (+)RNAs. All cDNAs were subjected to PCR using the KOD -Plus- Neo (Toyobo, Osaka, Japan) with primers (Forward: 5'-TCGTCGGCAGCGTCAGATGTGTATAAGAGACACACGGAAAGATGACAGACATTGACATTG-3', and Reverse: 5'-GTCTCGTGGGCTCGGAGATGTGTATAAGAGACAGCATTCTCTCCTATGGTTGCCTCTTGC-3') under the following conditions: one initial cycle of 94 °C for 2 min; five cycles of 98 °C for 30 s, 55 °C for 30 s, 68 °C for 30 s; and twenty cycles of 98 °C for 30 s, 65 °C for 30 s, 68 °C for 30 s. The PCR products were subjected to agarose gel electrophoresis and purified by using the Wizard SV Gel and PCR Clean-Up System (Promega, Madison, WI, USA).

**RT-PCR for RNA editing region of viral RNA in purified virions**

For the genome in rPIV2 virion, the Vero cells were infected with rPIV2^wt^ or rPIV2^IQ^ at an MOI of 0.1. At 48 hpi, the supernatants of Vero cells were centrifuged at 3,000 *g* for 5 min to remove cell debris, and then pelleted through a TNE (10 mM Tris-HCl [pH 7.4], 150 mM NaCl, 1 mM EDTA)-25% glycerol cushion for 20 min at 20,000 *g* at 4°C. The RNA in the pellet was extracted by using Isogen II. To increase the efficiency of RNA extraction, Dr.GenTLE precipitation carrier (Takara) was used according to the manufacturer's instructions. cDNA synthesis, PCR amplification and purification of PCR products were performed as shown above. We have confirmed that there were few amplifications of antigenome and mRNA in the purified virion, thus only genome amplicon was subjected for sequencing.

**Library preparation and Illumina sequencing**

Libraries were prepared by attaching dual index and Illumina sequencing adapters to the purified PCR products, using the Nextera XT Index Kit V2. The procedure followed 16S Metagenomic Sequencing Library Preparation Part #15044223 Rev. B with limited-cycle amplification (https://support.illumina.com/documents/documentation/chemistry_documentation/16s/16s-metagenomic-library-prep-guide-15044223-b.pdf). The product was checked for quality using an Agilent Technologies 2100 Bioanalyzer and a DNA 1000 chip to verify the template size distribution, in accordance with the Illumina qPCR Quantification Protocol Guide. The library was subjected to paired-end (2 × 300 bp) sequencing on the Illumina MiSeq system (Macrogen, Seoul, South Korea).

**Calculation of RNA editing efficiency**

To calculate RNA editing efficiency in mRNA, antigenome and genome, read sequences of each RNA-seq data were mapped to the hPIV2 reference genome (accession no. AB176531) by the Magic-Blast under the default condition (version. 1.5.0) (https://doi.org/10.1186/s12859-019-2996-x). The number of G insertions at RNA editing site was counted by a custom script.

**Quantitative real-time RT-PCR (RT-qPCR**

Total RNAs were isolated from rPIV2-infected cells using Isogen II according to the manufacturer’s instructions. cDNA synthesis was carried out using a PrimeScript RT reagent kit (Takara) with oligo-dT primer for all mRNAs including readthrough mRNAs. The qPCR was performed by using Brilliant III Ultra-Fast SYBR Green qPCR Master Mix (Agilent Technologies, Santa Clara, CA, USA) under the following conditions: one initial cycle of 95 ˚C for 30 s; 40 cycles of 95 ˚C for 5 s and 60 ˚C for 30 s. The primers for NP, P, M, and F mRNAs were previously described (2). The primers used for readthrough mRNAs were: NP-P readthrough mRNA, Forward, 5′-GACATTTCTCTTGAGCTAGTTGGAG-3′, and Reverse, 5′- TGGTGTATGTTGGTTCCTCGG-3′; P-M readthrough mRNA, Forward, 5′- AAACGGTCAATAATACGCTCTGCA-3′, and Reverse, 5′- GGGTGAAGTTGGATCTGCTGG-3′; M-F readthrough mRNA, Forward, 5′- ATCAATTCCAAACTTGGAAGCCATC-3′, and Reverse, 5′- CTATCATTGGATGCAGGTGATGC-3′. A standard curve was generated from dilutions whose copy numbers were known, and the RNA in samples per well was quantified based on this standard curve.

**Data availability**

All study data are included in the article and/or supporting information. The dataset of amplicon sequencing is available in the DNA Data Bank of Japan (DDBJ) under Run accession numbers DRR524078–DRR524095 and DRR668219–DRR668224.

**References**

1. Saka N, Matsumoto Y, Ohta K, Kolakofsky D, Nishio M. 2022. A Point Mutation in the Human Parainfluenza Virus Type 2 Nucleoprotein Leads to Two Separate Effects on Virus Replication. J Virol. 96:e0206721.

2. Matsumoto Y, Ohta K, Goto H, Nishio M. 2016. Parainfluenza virus chimeric mini-replicons indicate a novel regulatory element in the leader promoter. J Gen Virol. 97(7):1520–1530.
